# Effect of fumigation height and time on cryopreservation of ram semen

**DOI:** 10.1038/s41598-024-61947-x

**Published:** 2024-05-13

**Authors:** Liuming Zhang, Xuyang Wang, Caiyu Jiang, Yuxuan Sun, Tariq Sohail, Xiaomei Sun, Jian Wang, Yongjun Li

**Affiliations:** Key Laboratory for Animal Genetics & Molecular Breeding of Jiangsu Province, College of Animal Science and Technology, Yangzhou University, Yangzhou, 225009 China

**Keywords:** Fumigation height and time, Ram, Spermatozoa, Motility parameters, Functional integrity, ROS, Zoology, Animal physiology

## Abstract

The cooling rate is a crucial factor in the process of freezing semen, influencing the overall freezing effectiveness. The height and time of fumigation can significantly impact the rate of cooling. Appropriate cooling rates can help minimize the formation of ice crystals in spermatozoa and reduce potential damage to them. Therefore, the aim of this study was to evaluate the effect of different fumigation heights and time for the cryopreservation of Hu ram semen. Experiments I–IV assessed the effect of semen cryopreservation by testing the post-thawed spermatozoa total motility (TM), progressive motility (PM) and kinetic parameters fumigated at distances of 2, 4, 6 and 8 cm for durations of 5, 10, 15 and 20 min, respectively. Based on the results of experiments I to IV, experiment V evaluated the effect of semen cryopreservation by testing the post-thawed spermatozoa TM, PM, kinetic parameters, plasma membrane integrity, acrosome integrity and reactive oxygen species (ROS) level fumigated at distances of 2, 4, 6 and 8 cm for duration of 20 min. The results indicated that fumigation at 2 cm for 20 min significantly (*P* < 0.05) improved spermatozoa TM, PM, mean angular displacement (MAD), plasma membrane integrity and acrosome integrity compared to other groups. Additionally, it significantly (*P* < 0.05) reduced spermatozoa ROS level compared to the 6 and 8 cm groups. In conclusion, fumigation for 20 min at a distance of 2 cm from the liquid nitrogen surface is the most suitable cooling method for the cryopreservation of Hu ram semen.

## Introduction

Rearing Hu sheep is gaining popularity in China due to their high fertility rates and tendency for multiple births^1^. Farmers want to increase production through the selective breeding. Therefore, the use of semen from Hu rams with superior genetics through artificial insemination (AI) is an efficient tool. AI could enhance livestock breeding, particularly for intensive, large-scale and specialized productions^2–4^. Successful AI depends on the collection, preservation and utilization of semen^5^. The most important part is the preservation of semen^6^. Liquid and solid storage are commonly utilized for semen preservation.

Although spermatozoa stored in a liquid state have high motility, the effective storage time of semen is too short to meet the requirements, which significantly hinders the application of AI technology^7^. Semen cryopreservation has enhanced herd breeding by facilitating cross-regional exchanges of genetic resources through the promotion of in vitro storage and long-distance transportation^8^. Although the spermatozoa motility will decrease after thawing, it can theoretically be preserved permanently. However, ram spermatozoa is very sensitive to freezing compared to other species because of spermatozoa membrane containing too much polyunsaturated fatty acids (PUFAs)^9,10^. Therefore, in order to minimize the impact of freezing shock, it is essential to establish an appropriate freezing protocol for ram semen.

The success of semen cryopreservation techniques depends on the diluent, freezing and thawing protocols^11,12^. The cooling rate is crucial in the semen freezing protocol. Additionally, the height and time of fumigation can significantly affect the cooling rate. When the cooling rate is too slow, the water molecules inside and outside the spermatozoa cell will rearrange in a geometric pattern to form ice crystals, and the slower the cooling rate, the larger the ice crystals formed^13^. With the formation, expansion and movement of ice crystals, two types of damage can occur: physical and mechanical damage, and chemical solution damage. The first is the rupture of cell membranes, damage to organelles, destruction of chromosomes and enzyme structures, causing mechanical damage and spermatozoa death^14,15^. Secondly, when ice crystals form, the solute concentration and osmotic pressure in spermatozoa cells will increase, and water molecules permeate from the inside to the outside of spermatozoa cells, causing continuous contraction and dehydration of spermatozoa cells, which will lead to the damage of cell membrane structure and protein degeneration^16,17^. Therefore, ice crystals are the main factor causing spermatozoa death, and appropriate cooling rate can help avoid the production of ice crystals in spermatozoa as much as possible and reduce the damage to spermatozoa^18^.

Based on the above information, the aim of this study was to investigate the effect of fumigation height and time on the cryopreservation of ram spermatozoa and to enhance the freezing protocol by evaluating post-thawed spermatozoa motility parameters, kinetic parameters, plasma membrane integrity, acrosome integrity and reactive oxygen species (ROS) level.

## Results

### Effect of fumigation for 5, 10, 15 and 20 min at the height of 2 cm on spermatozoa motility and kinetic parameters during cryopreservation

As shown in Table 1, the post-thawed spermatozoa TM of the 15 and 20 min fumigation groups was significantly higher (*P* < 0.05) than 5 min group, but it was not significantly higher (*P* > 0.05) than 10 min group. The post-thawed spermatozoa PM of the 15 and 20 min fumigation groups was significantly higher (*P* < 0.05) than that of the 5 and 10 min groups and the 15, 20 min groups were not significantly (*P* > 0.05) different from each other. The post-thawed spermatozoa VSL of the 20 min fumigation group was significantly higher (*P* < 0.05) than 15 min group. Additionally, these groups did not show significantly (*P* > 0.05) differences from each other in terms of spermatozoa VCL, VAP, ALH and MAD.

**Table 1:** Effect of different fumigation time at the height of 2 cm on spermatozoa motility and kinetic parameters during cryopreservation.

Fumigation time (min)	TM (%)	PM (%)	VSL (µm/s)	VCL (µm/s)	VAP (µm/s)	ALH (µm)	MAD (°/s)
5	77.80 ± 0.33^b^	63.48 ± 0.61^b^	36.77 ± 0.55^a^	58.82 ± 1.43	41.59 ± 1.01	17.23 ± 0.42	53.58 ± 0.51
10	80.40 ± 0.96^ab^	64.33 ± 1.25^b^	35.48 ± 0.69^ab^	55.84 ± 1.85	39.49 ± 1.31	16.36 ± 0.54	55.62 ± 4.43
15	81.13 ± 0.87^a^	67.90 ± 0.59^a^	33.94 ± 0.75^b^	53.69 ± 0.80	37.96 ± 0.57	15.73 ± 0.23	60.42 ± 4.30
20	82.28 ± 1.36^a^	68.40 ± 0.68^a^	36.87 ± 0.61^a^	57.84 ± 1.73	40.90 ± 1.22	16.94 ± 0.51	65.80 ± 4.21

Different lowercase superscripts in the same column show significant differences (*P* < 0.05).

### Effect of fumigation for 5, 10, 15 and 20 min at the height of 4 cm on spermatozoa motility and kinetic parameters during cryopreservation

As shown in Table 2, the post-thawed spermatozoa PM of the 5 min fumigation group was significantly lower (*P* < 0.05) than in the other groups, but there was no significant difference (*P* > 0.05) in the 10, 15 and 20 min groups. Additionally, these groups did not show significant (*P* > 0.05) differences from each other in terms of spermatozoa TM, VSL, VCL, VAP, ALH and MAD.

**Table 2:** Effect of different fumigation time at the height of 4 cm on spermatozoa motility and kinetic parameters during cryopreservation.

Fumigation time (min)	TM (%)	PM (%)	VSL (µm/s)	VCL (µm/s)	VAP (µm/s)	ALH (µm)	MAD (°/s)
5	61.10 ± 2.25	43.21 ± 0.73^b^	37.36 ± 0.61	56.78 ± 0.89	40.15 ± 0.63	16.63 ± 0.26	38.85 ± 2.23
10	66.80 ± 1.45	47.73 ± 0.76^a^	38.13 ± 1.44	59.01 ± 0.85	41.73 ± 0.60	17.28 ± 0.25	42.19 ± 4.63
15	63.82 ± 0.94	47.03 ± 1.27^a^	38.71 ± 2.06	58.44 ± 1.76	41.33 ± 1.24	17.12 ± 0.51	43.75 ± 2.67
20	65.95 ± 1.67	48.08 ± 1.09^a^	38.43 ± 0.97	56.86 ± 1.00	40.21 ± 0.71	16.65 ± 0.30	35.48 ± 0.91

Different lowercase superscripts in the same column show significant differences (*P* < 0.05).

### Effect of fumigation for 5, 10, 15 and 20 min at the height of 6 cm on spermatozoa motility and kinetic parameters during cryopreservation

The post-thawed spermatozoa TM was significantly higher (*P* < 0.05) in the 20 min fumigation group compared with the 5 min group, but it was not significantly higher (*P* > 0.05) than the 10, 15 min groups as shown in Table 3. The post-thawed spermatozoa PM and MAD were significantly higher (*P* < 0.05) in the 20 min fumigation group compared with the other groups. Additionally, the post-thawed spermatozoa VSL, VCL, VAP and ALH in the 20 min fumigation group were the highest, but they did not show a significant difference (*P* > 0.05) compared to the other groups.

**Table 3:** Effect of different fumigation time at the height of 6 cm on spermatozoa motility and kinetic parameters during cryopreservation.

Fumigation time (min)	TM (%)	PM (%)	VSL (µm/s)	VCL (µm/s)	VAP (µm/s)	ALH (µm)	MAD (°/s)
5	68.68 ± 0.55^b^	51.55 ± 0.44^b^	39.07 ± 0.93	60.73 ± 2.50	42.94 ± 1.77	17.79 ± 0.73	48.08 ± 2.91^b^
10	71.53 ± 2.28^ab^	54.39 ± 2.21^b^	39.83 ± 1.26	60.05 ± 1.41	42.46 ± 1.00	17.59 ± 0.42	44.58 ± 1.25^b^
15	70.60 ± 1.08^ab^	54.42 ± 1.20^b^	38.81 ± 0.83	60.38 ± 0.62	42.69 ± 0.44	17.68 ± 0.18	47.81 ± 3.03^b^
20	74.52 ± 0.35^a^	61.37 ± 1.23^a^	40.76 ± 1.33	64.03 ± 0.98	45.28 ± 0.69	18.75 ± 0.29	57.46 ± 1.77^a^

Different lowercase superscripts in the same column show significant differences (*P* < 0.05).

### Effect of fumigation for 5, 10, 15 and 20 min at the height of 8 cm on spermatozoa motility and kinetic parameters during cryopreservation

The post-thawed spermatozoa PM of the 20 min fumigation group was significantly higher (*P* < 0.05) than in other groups, but it did not show a significant difference (*P* > 0.05) in the 5, 10 and 15 min groups as shown in Table 4. The post-thawed spermatozoa TM, VSL, VCL, VAP, ALH and MAD of the 20 min fumigation group were the highest, but they were not significantly higher (*P* > 0.05) than those of the other groups.

**Table 4:** Effect of different fumigation time at the height of 8 cm on spermatozoa motility and kinetic parameters during cryopreservation.

Fumigation time (min)	TM (%)	PM (%)	VSL (µm/s)	VCL (µm/s)	VAP (µm/s)	ALH (µm)	MAD (°/s)
5	62.59 ± 1.11	42.40 ± 1.07^b^	35.62 ± 1.40	54.06 ± 1.84	38.22 ± 1.30	15.83 ± 0.54	34.37 ± 3.06
10	60.44 ± 2.45	41.83 ± 1.06^b^	35.95 ± 1.81	54.33 ± 2.01	38.42 ± 1.42	15.91 ± 0.59	38.00 ± 1.93
15	59.12 ± 1.81	43.24 ± 1.23^b^	35.42 ± 0.73	53.07 ± 0.57	37.53 ± 0.40	15.54 ± 0.16	35.45 ± 2.46
20	64.15 ± 2.09	46.87 ± 1.03^a^	36.25 ± 1.49	55.02 ± 1.66	38.90 ± 1.18	16.11 ± 0.49	40.94 ± 3.00

Different lowercase superscripts in the same column show significant differences (*P* < 0.05).

### Effect of fumigation for 20 min at the height of 2, 4, 6 and 8 cm on spermatozoa motility and kinetic parameters during cryopreservation

As shown in Table 5, the post-thawed spermatozoa TM and PM were significantly higher (*P* < 0.05) in the 2 cm fumigation group compared with the other groups. And the post-thawed spermatozoa TM and PM did not show a significant difference (*P* > 0.05) in the 4, 6 and 8 cm groups. The post-thawed spermatozoa MAD of the 2 cm fumigation group was significantly higher (*P* < 0.05) than in the 4 and 8 cm groups, but it did not show a significant difference (*P* > 0.05) compared to the 6 cm group. Additionally, these groups did not show significant (*P* > 0.05) differences in spermatozoa VSL, VCL, VAP and ALH.

**Table 5:** Effect of fumigation for 20 min at the different height on spermatozoa motility and kinetic parameters during cryopreservation.

Fumigation height (cm)	TM (%)	PM (%)	VSL (µm/s)	VCL (µm/s)	VAP (µm/s)	ALH (µm)	MAD (°/s)
2	78.33 ± 1.12^a^	63.95 ± 1.03^a^	42.06 ± 1.54	64.31 ± 2.57	45.48 ± 1.81	18.84 ± 0.75	55.18 ± 2.00^a^
4	70.78 ± 1.04^b^	54.30 ± 1.06^b^	39.99 ± 0.68	59.16 ± 0.74	41.83 ± 0.52	17.33 ± 0.22	44.16 ± 2.29^b^
6	70.42 ± 2.51^b^	53.66 ± 1.86^b^	39.17 ± 0.58	59.36 ± 1.16	41.97 ± 0.82	17.38 ± 0.34	46.42 ± 2.33^ab^
8	68.37 ± 3.13^b^	52.70 ± 1.67^b^	39.51 ± 1.15	59.52 ± 1.02	42.09 ± 0.72	17.43 ± 0.30	45.01 ± 4.07^b^

Different lowercase superscripts in the same column show significant differences (*P* < 0.05).

### Effect of fumigation for 20 min at the height of 2, 4, 6 and 8 cm on spermatozoa plasma membrane integrity during cryopreservation

**Figure 1:**
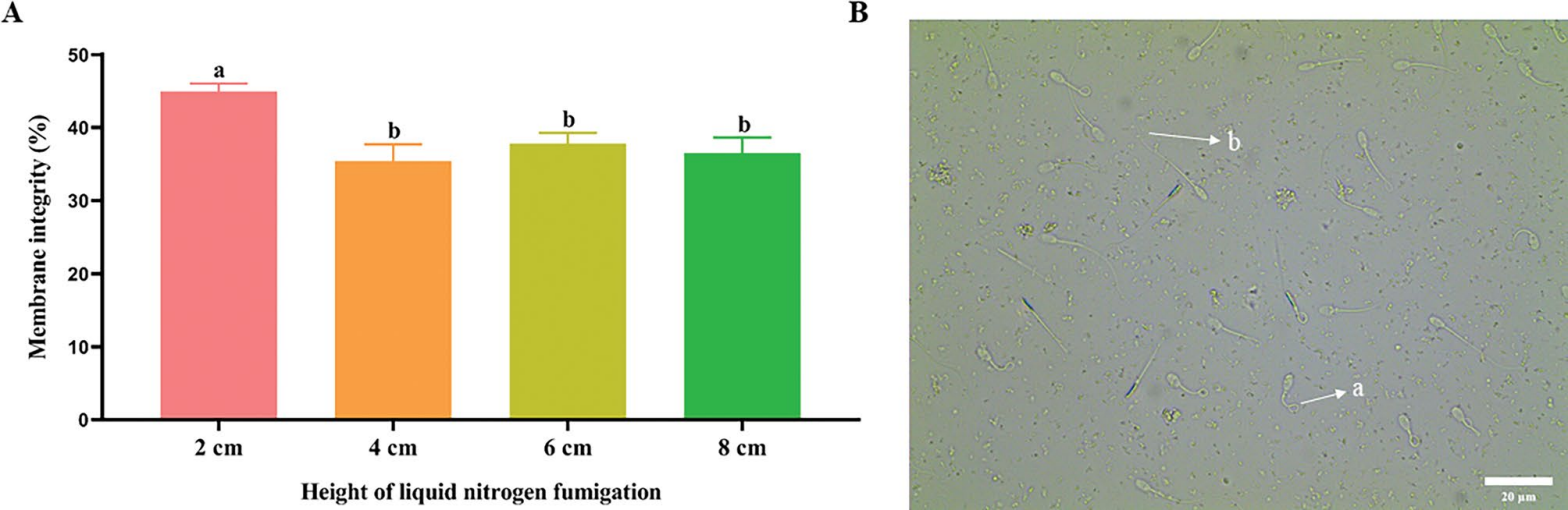
Detection of the spermatozoa plasma membrane. (**A**) Effect of fumigation for 20 min at the different height on spermatozoa plasma membrane integrity during cryopreservation. Values with different lowercase letters indicate difference (*P* < 0.05) among groups. (**B**) Morphology of curly tail of spermatozoa in HOST. (a) Spermatozoa with intact membrane. (b) Spermatozoa with damaged membrane.

As shown in Fig. 1A, the post-thawed spermatozoa plasma membrane integrity of the 2 cm fumigation group was significantly higher (*P* < 0.05) than that of the other groups, but it did not show a significant difference (*P* > 0.05) in the 4, 6, 8 cm groups. The type of tail curls (a) indicate intact-membrane spermatozoa, while the non-curl type (b) represents the spermatozoa with a damaged membrane as shown in Fig. 1B.

### Effect of fumigation for 20 min at the height of 2, 4, 6 and 8 cm on spermatozoa acrosome integrity during cryopreservation

**Figure 2:**
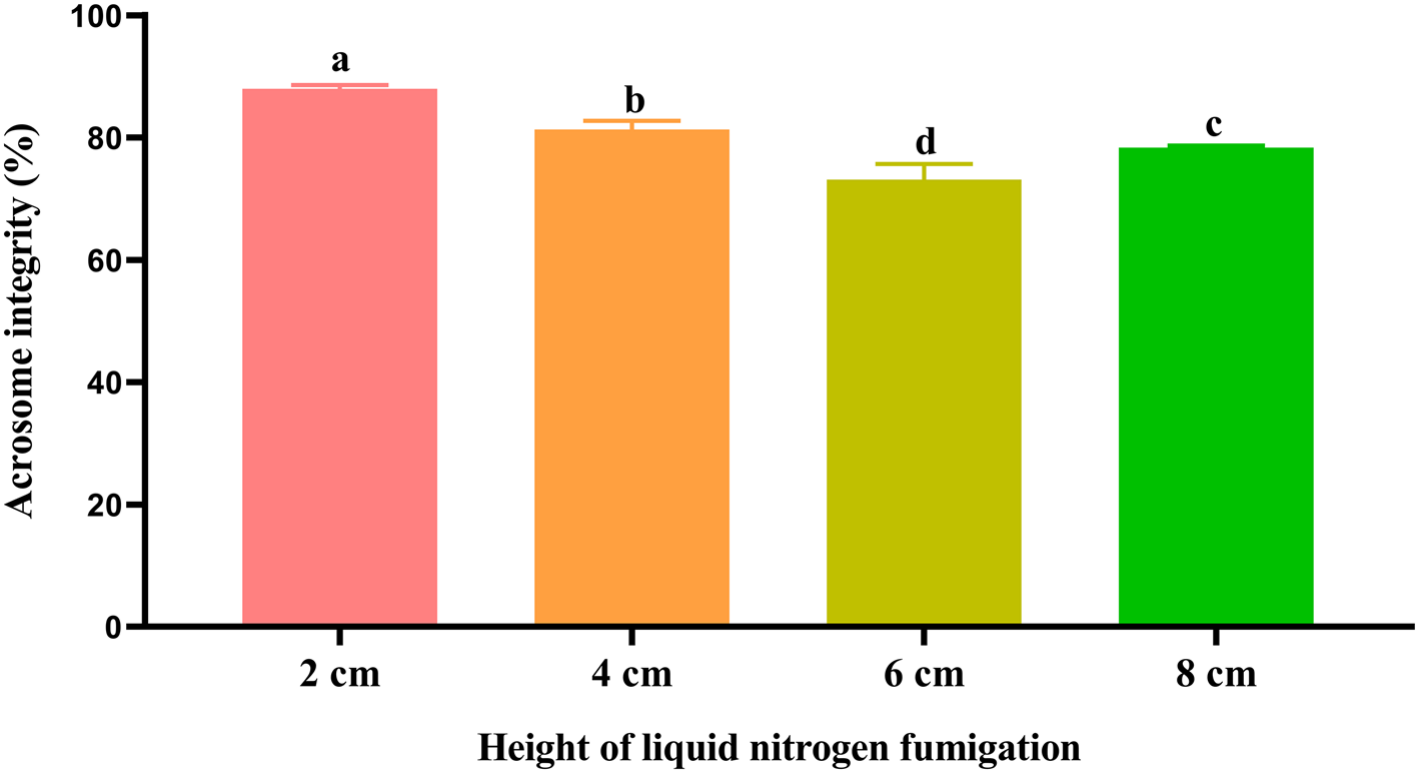
Effect of fumigation for 20 min at the different height on spermatozoa acrosome integrity during cryopreservation. Values with different lowercase letters indicate difference (*P* < 0.05) among groups.

The post-thawed spermatozoa acrosome integrity of the 2 cm fumigation group was significantly higher (*P* < 0.05) than in the other groups as shown in Fig. 2. Additionally, the post-thawed spermatozoa acrosome integrity of the 4 cm fumigation group was significantly higher (*P* < 0.05) than in the 6 and 8 cm groups. The post-thawed spermatozoa acrosome integrity of the 6 cm fumigation group was significantly lower (*P* < 0.05) than in the other groups.

### Effect of fumigation for 20 min at the height of 2, 4, 6 and 8 cm on spermatozoa ROS level during cryopreservation

**Figure 3:**
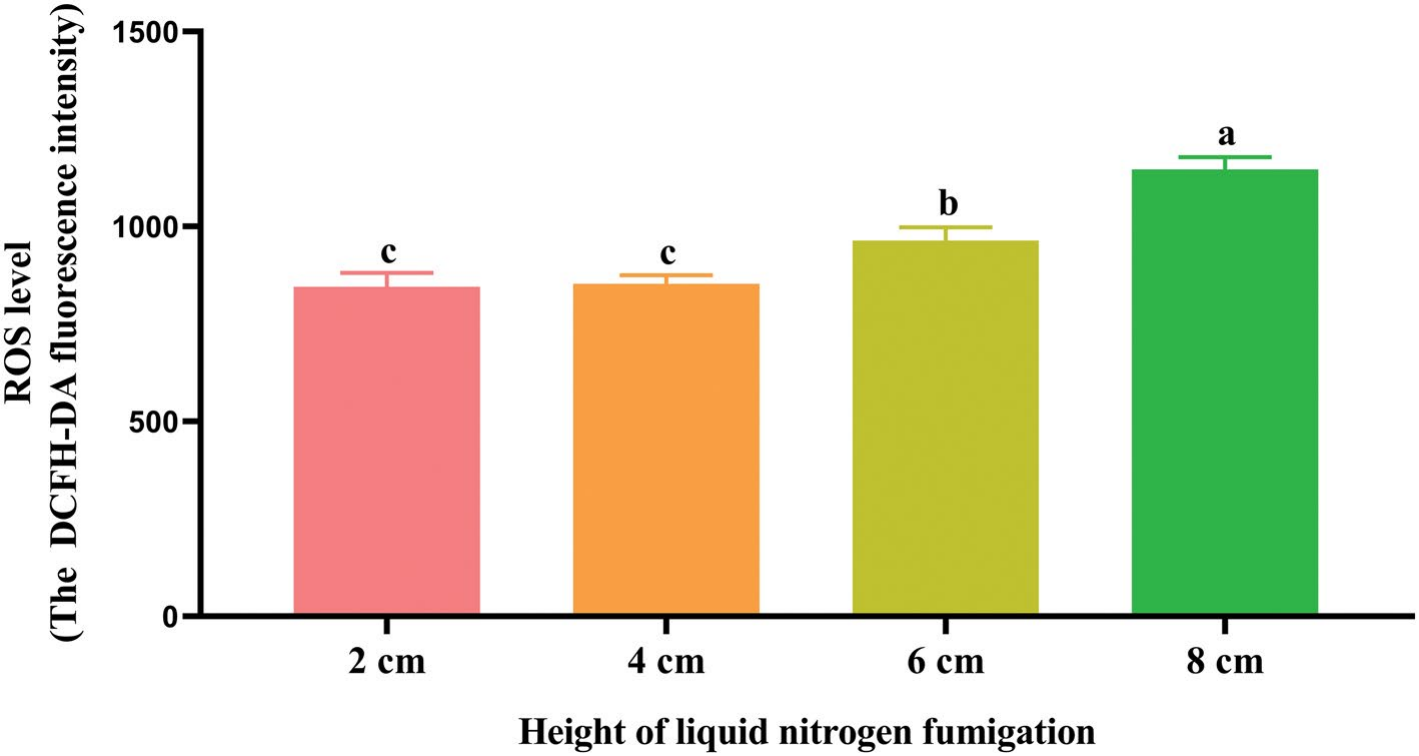
Effect of fumigation for 20 min at the different height on spermatozoa ROS level during cryopreservation. Values with different lowercase letters indicate difference (*P* < 0.05) among groups.

As shown in Fig. 3, the post-thawed spermatozoa ROS level was significantly lower (*P* < 0.05) in the 2 and 4 cm fumigation groups compared with the 6 and 8 cm groups. After thawing, the spermatozoa ROS level of the 8 cm fumigation group was higher (*P* < 0.05) than in the other groups.

## Discussion

Semen equilibration, freezing and thawing are three important stages in the semen freezing process, each playing a crucial role in the effectiveness of semen cryopreservation. The cooling rate in the process of freezing semen is the key factor affecting the freezing effect of semen^19^. Ram spermatozoa are very sensitive to temperature changes. Although diluted semen can reduce the damage caused by low-temperature shock, the cooling rate should not be too fast, otherwise it will easily cause cold shock, leading to spermatozoa death^20^. At the same time, if the cooling rate is too slow, it may also be harmful due to the inability of spermatozoa to pass through the dangerous temperature range (0 to − 60 °C) quickly, leading to damage from ice crystal formation^21^. Liquid nitrogen fumigation and program-controlled cryostat freezing are the primary methods used for semen freezing currently. For most laboratories dealing with non-industrialized species and production, liquid nitrogen fumigation remains the primary freezing method^22,23^. The cooling rate of liquid nitrogen fumigation is determined by the height from the liquid nitrogen surface. Therefore, the fumigation height and time during semen freezing should be controlled within a specific range.

In semen freezing, even though antifreeze is added to the diluent, it may still be affected by dangerous temperature ranges^24^. In this study, experiment I to IV found that the post-thawed spermatozoa PM after fumigation for 20 min at all fumigation heights was significantly higher than that after fumigation for 5 min. This may be due to the fact that the fumigation time is too short for the semen to reach an appropriate temperature before being directly placed into liquid nitrogen, leading to a decrease in spermatozoa motility^25^. This is consistent with Jha’s^26^ research findings, the sudden drop in temperature leads to cryogenic damage to spermatozoa.

In this study, as shown in Table 5, the post-thawed spermatozoa motility parameters and MAD in the 2 cm fumigation group for 20 min were significantly higher than those in other groups. This may be due to the higher cooling rate at 2 cm, which allows spermatozoa to pass through the dangerous area faster, reducing the production of ice crystals, and thus minimizing spermatozoa damage^27^. At the same time, the research shows that the cooling rate of semen samples at a distance of 2.5 cm from the liquid nitrogen surface can reach 65 °C/min^28^. The study by Kucuk^29^ on frozen semen of Saanen goats showed that fumigation with 4 cm for 7 min could achieve a good freezing effect. Motamedi-Mojdehi^30^ found that spermatozoa motility was higher after fumigation at a distance of 4.5 cm for 13 min in the study of Taleshi ram semen. Falchi’s^31^ research on bull spermatozoa showed that fumigation with 5 cm for 7 min could achieve a better semen freezing effect. However, it was found in this study that the quality of post-thawed spermatozoa quality after fumigation at 2 cm was higher, which contrasts with the findings of previous research. This difference may be attributed to variances in spermatozoa resistance to freezing, which could be influenced by species or breeds, as well as variances in the composition of the diluent used and the pre-equilibration time^32,33^. Another study on human spermatozoa have shown that differences in the volume of straws can also affect semen freezing^34^.

The appropriate cooling rate plays a crucial role in maintaining the structural integrity of spermatozoa^35^. In this study, the integrity of spermatozoa plasma membrane and acrosome in the group fumigated at a distance of 2 cm for 20 min was significantly higher than in the other groups. This may be due to the fact that the other groups were farther away from the liquid nitrogen surface, reaching 4, 6 and 8 cm, which leads to slow cooling rate, excessive cell contraction and destruction of the spermatozoa structural integrity^36^. This is because ice crystals begin to form in the straws when the temperature reaches − 7 °C. At this point, solid–liquid coexistence occurs, with the concentration in the solution increasing, and the water in the spermatozoa is gradually being transported to the solution^37^. Therefore, if the spermatozoa remain in this hypertonic environment for an extended period, they will experience excessive dehydration and chemical damage due to high ion concentration^38^. Morris’s^39^ research on Fell Pony stallion semen similarly demonstrated that the osmotic pressure between the diluent and spermatozoa during freezing can lead to a significant decline in the effectiveness of freezing. The change in osmotic pressure destabilizes the spermatozoa plasma membrane, affecting the balance of Ca^2+^ and acrosome integrity^40^.

Appropriate level of ROS play a crucial role in spermatozoa capacitation, acrosome reaction, and the preservation of cell membrane fluidity^41,42^. However, when the level of ROS exceeds a certain threshold, it can have a detrimental effect, leading to oxidative stress and damage to spermatozoa^43^. In this study, the level of ROS in post-thawed spermatozoa in the 2 cm fumigation group for 20 min was significantly lower than that in the 6 and 8 cm groups. Ball’s^44^ study demonstrated that the ROS level gradually increased with the decrease of semen quality after thawing. Meanwhile, Aitken^45^ obtained the same result in the study of horse spermatozoa, where the ROS level gradually increased as sperm motility decreased. This study found that the quality of post-thawed spermatozoa quality in the 2 cm fumigation group for 20 min was significantly improved, similar to the results of this study. The liquid nitrogen fumigation cooling method has the advantages of simple operation and low cost. Therefore, the purpose of this study is to enhance the technology of ram semen cryopreservation, improve the efficacy of semen cryopreservation, and offer insights for its application in production practices and future theoretical research.

In summary, the results indicated improved post-thawed spermatozoa motility parameters, kinetic parameters, functional integrity, and decreased post-thawed spermatozoa ROS level of Hu ram spermatozoa frozen at a distance of 2 cm for 20 min. Fumigation for 20 min at a distance of 2 cm from the liquid nitrogen surface is the most suitable cooling method for the cryopreservation of Hu ram semen.

## Materials and methods

### Ethics statement

The experimental protocols were approved by the Animal care committee of the Yangzhou University (ID: 202206132). All methods were carried out in accordance with relevant guidelines and regulations. All methods are reported in accordance with ARRIVE guidelines.

### Media and reagents

Tris, glucose, citric acid and glycerol were purchased from Sangon Biotech (Shanghai, China). Penicillin–streptomycin was purchased from Thermo Fisher (Waltham, Massachusetts, USA). The freezing extender I contained Tris base 300.48 mM, glucose 27.75 mM, citric acid 94.72 mM and penicillin–streptomycin 200 IU/mL with 20% egg yolk. The freezing extender II contained 94% freezing extender I and 6% glycerol. Peanut agglutinin conjugated with fluorescein isothiocyanate (FITC‐PNA) was purchased from Sigma (St Louis, MO, USA). Propidine iodide (PI) was purchased from Solarbio (Beijing, China). ROS assay kit was purchased from Beyotime (Shanghai, China).

### Experimental layout

Several experiments were conducted in this study to explore the optimal time for fumigation at various heights. In the first experiment, the impact of fumigation for 5, 10, 15 and 20 min at a height of 2 cm on semen cryopreservation was investigated by assessing the total motility (TM), progressive motility (PM) and kinetic parameters of thawed spermatozoa. Experiment II investigated the impact of fumigation for 5, 10, 15 and 20 min at a height of 4 cm on semen cryopreservation by analyzing the parameters mentioned above. Experiment III investigated the impact of fumigation for 5, 10, 15 and 20 min at a height of 6 cm on semen cryopreservation by analyzing the aforementioned parameters. Experiment IV investigated the impact of fumigation for 5, 10, 15 and 20 min at a height of 8 cm on semen cryopreservation by analyzing the above parameters mentioned above. Based on the results, experiment V was designed to evaluate the optimum fumigation height under the condition of 20 min fumigation time by detecting the TM, PM, kinetic parameters, plasma membrane integrity, acrosome integrity and ROS level after post-thaw.

### Animals and semen collection

The five rams were kept at the sheep facility located at the University of Yangzhou. The sheep were fed in accordance with the regulations of the experimental sheep farm. The rams were fed 0.7 kg of concentrate per day and had ad libitum access to hay and water. The experiment was carried out from March to May 2023. All animal procedures conform to the requirements of the Animal Ethics Committee of Yangzhou University (Approval ID: 202206132). A total number of 75 ejaculates (15 ejaculates per ram) were collected three times a week from five rams using an artificial vagina (SJ020, Muqi, Shanghai, China). The semen was stored in a 37 °C thermos and the samples were transported back to the laboratory within 30 min. The semen sample volume (mL) was measured using a micropipette. The spermatozoa concentration and total motility were assessed using a computer-assisted spermatozoa analyzer (CASA, ML-608JZ II Mailang, Nanning, China). The spermatozoa morphology was evaluated using eosin-nigrosin staining and spermatozoa abnormalities were classified into head, middle or tail defects according to the type of defects. The normal semen standards used were as follows: a volume range of 0.5 to 1.5 mL; spermatozoa concentration ≥ 2.5 × 10^9^ spermatozoa/mL; total motility ≥ 80%; abnormal morphology ≤ 15%. Qualified semen was pooled together to eliminate individual differences.

### Cryopreservation and thawing of spermatozoa

Firstly, semen samples were rediluted with freezing extender I to a ratio of 1:2 and then were equilibrated at 4 °C for 2.5 h. Secondly, samples were rediluted with freezing extender II to a ratio of 1:1 and then also were equilibrated at 4 °C for 2.5 h. Immediately, all semen samples were loaded into 0.25 mL French straws (IMV, L’Aigle, France) with a self-made syringe. Filled straws were held 2–8 cm above liquid nitrogen for different time (5–20 min) and then plunged. Samples were stored for at least one week, thawed at 70 °C in a water bath for 5 s, and then used for further investigation and analyses.

### Evaluation of spermatozoa motility and kinematic characteristics

Motility and kinematic characteristics were analyzed using a CASA. Briefly, an aliquot of thawed semen was diluted in freezing extender I without egg yolk (1:9) and 1.4 µL was placed on a warmed (37 °C) MACRO spermatozoa counting chamber (YA-1, Yucheng, Nanjing, China) and loaded into the system. Each sample was analyzed in four microscope (ML-800, Mailang, Nanning, China) fields for the following parameters: Total motility (TM, %), Progressive motility (PM, %), Straight line velocity (VSL, μm/s), Curvilinear velocity (VCL, μm/s), Average path velocity (VAP, μm/s), mean angular displacement (MAD, °/s) and Amplitude of lateral head displacement (ALH, μm).

### Assessment of plasma membrane integrity

The hypotonic swelling test (HOST) was used to assess the integrity of the plasma membrane of thawed spermatozoa. HOST is based on the resistance of the spermatozoa membrane to stress conditions in a hypotonic solution (108 m Osm/L, consisting of 0.0049 g/mL sodium citrate and 0.009 g/mL fructose dissolved in water). Briefly, an aliquot of thawed semen was diluted in freezing extender I without egg yolk (1:2) and 20 µL was mixed with 200 µL hypotonic solution. After 30 min incubation at 37 °C in a water bath, spermatozoa were examined using a phase-contrast microscope (CX31, Olympus, Tokyo, Japan) at a magnification of × 400 and at least 200 spermatozoa with swollen and non-swollen tails were recorded.

### Assessment of acrosome integrity

Spermatozoa acrosome integrity was assessed by differential staining with PI and FITC-PNA. Briefly, an aliquot of thawed semen was diluted in freezing extender I without egg yolk (1:12) and 100 µL was mixed with 2 µL PI (0.5 mg/mL) and 2 µL FITC-PNA (200 µg/mL). After 30 min incubation at 37 °C in the dark, add 700 µL PBS to the sample and examine using a FACS calibur flow cytometer for cell analysis (Beckman Coulter, Shanghai, China). The integrity of the acrosome was characterized in the FITC^−^/PI^+^ and FITC^-^/PI^-^ quadrants. For each sample, 10,000 spermatozoa were considered.

### Assessment of ROS level

Spermatozoa ROS level was assessed using a fluorescent probe with DCFH-DC^46^. Briefly, an aliquot of thawed semen was diluted in freezing extender I without egg yolk (1:12) and 50 µL were mixed with 1 µL DCFH-DC (10 mM). After 30 min incubation at 37 °C in the dark, add PBS to wash the sample. The sample was added to 400 µL PBS and analyzed using a multifunctional microplate reader (PerkinElmer, Waltham, MA, USA) at excitation/emission = 488/525 nm and the ROS level was determined based on the fluorescence intensity.

### Statistical analysis

The data obtained in the experiment were analyzed using Statistical Package for the Social Sciences (SPSS, IBM, version 25.0). The Shapiro–Wilk normality analysis was performed to detect whether the data conforms to the normal distribution. Semen with respect to TM, PM, kinematic characteristics, plasma membrane integrity, acrosome integrity and ROS level were compared using Duncan test by one-way analysis of variance (ANOVA) procedures because the data show normal distribution. Results were reported as the mean ± SEM and the *P* < 0.05 was considered to be significant.

## Data Availability

The original contributions presented in the research are included in the manuscript.

## References

[1] Li X, Yao X, Bao Y, Deng K, Deng M, Yang F, Sun X, You P, Cai Q, Wang F (2023). PPP2R2A promotes testosterone secretion in Hu sheep Leydig cells via activation of the AKT/mTOR signaling pathway. J. Mol. Endocrinol..

[2] Zuidema D, Kerns K, Sutovsky P (2021). An exploration of current and perspective semen analysis and sperm selection for livestock artificial insemination. Animals.

[3] Wiebke M, Hensel B, Nitsche-Melkus E, Jung M, Schulze M (2022). Cooled storage of semen from livestock animals (part I): Boar, bull, and stallion. Anim. Reprod. Sci..

[4] Washaya S, Tavirimirwa B, Dube S, Sisito G, Tambo G, Ncube S, Zhakata X (2019). Reproductive efficiency in naturally serviced and artificially inseminated beef cows. Trop. Anim. Health Prod..

[5] Yeste M, Rodríguez-Gil JE, Bonet S (2017). Artificial insemination with frozen-thawed boar sperm. Mol. Reprod. Dev..

[6] Zhang L, Wang Y, Sohail T, Kang Y, Niu H, Sun X, Ji D, Li Y (2021). Effects of taurine on sperm quality during room temperature storage in Hu sheep. Animals.

[7] Gillan L, Maxwell WM, Evans G (2004). Preservation and evaluation of semen for artificial insemination. Reprod. Fertil. Dev..

[8] Mehdipour M, Daghigh Kia H, Nazari M, Najafi A (2017). Effect of lecithin nanoliposome or soybean lecithin supplemented by pomegranate extract on post-thaw flow cytometric, microscopic and oxidative parameters in ram semen. Cryobiology.

[9] Vozaf J, Makarevich AV, Balazi A, Vasicek J, Svoradova A, Olexikova L, Chrenek P (2021). Cryopreservation of ram semen: Manual versus programmable freezing and different lengths of equilibration. Anim. Sci. J..

[10] Ari UÇ, Kulaksiz R, Öztürkler Y (2011). Freezability of Tushin ram semen extended with goat or cow milk based extenders. Reprod. Domest. Anim..

[11] Tekin N (2006). Effects of different taurine doses and freezing rate on freezing of row semen. Ankara Univ. Vet. Fak..

[12] Pontbriand D, Howard JG, Schiewe MC, Stuart LD, Wildt DE (1989). Effect of cryoprotective diluent and method of freeze-thawing on survival and acrosomal integrity of ram spermatozoa. Cryobiology.

[13] Yeste M (2016). Sperm cryopreservation update: Cryodamage, markers, and factors affecting the sperm freezability in pigs. Theriogenology.

[14] Petrunkina AM, Gröpper B, Töpfer-Petersen E, Günzel-Apel AR (2005). Volume regulatory function and sperm membrane dynamics as parameters for evaluating cryoprotective efficiency of a freezing extender. Theriogenology.

[15] Mutalik S, Salian SR, Avadhani K, Menon J, Joshi H, Hegde AR, Kumar P, Kalthur G, Adiga SK (2014). Liposome encapsulated soy lecithin and cholesterol can efficiently replace chicken egg yolk in human semen cryopreservation medium. Syst. Biol. Reprod. Med..

[16] Said TM, Gaglani A, Agarwal A (2010). Implication of apoptosis in sperm cryoinjury. Reprod. Biomed. Online.

[17] Bagchi A, Woods EJ, Critser JK (2008). Cryopreservation and vitrification: Recent advances in fertility preservation technologies. Expert Rev. Med. Devices.

[18] Igbokwe AA, Iyasere OS, Sobayo RA, Iyasere S, Animashaun RI, Balogun FA, Aganran ZO, Fasola MO, Adedokun AD, Lakehinde OA, Lasisi SO, Suleiman MR, Iyiola JD, Daramola JO (2019). Comparative effect of slow and rapid freezing on sperm functional attributes and oxidative stress parameters of goat spermatozoa cryopreserved with tiger nut milk extender. Reprod. Domest. Anim..

[19] Barbas JP, Mascarenhas RD (2009). Cryopreservation of domestic animal sperm cells. Cell. Tissue Bank..

[20] Demyda-Peyrás S, Bottrel M, Acha D, Ortiz I, Hidalgo M, Carrasco JJ, Gómez-Arrones V, Gósalvez J, Dorado J (2018). Effect of cooling rate on sperm quality of cryopreserved Andalusian donkey spermatozoa. Anim. Reprod. Sci..

[21] Yoon SJ, Rahman MS, Kwon WS, Park YJ, Pang MG (2016). Addition of cryoprotectant significantly alters the epididymal sperm proteome. PLoS ONE.

[22] Yang CH, Wu TW, Cheng FP, Wang JH, Wu JT (2016). Effects of different cryoprotectants and freezing methods on post-thaw boar semen quality. Reprod. Biol..

[23] Pan JM, Zhu KC, Liu J, Guo HY, Liu BS, Zhang N, Xian L, Sun JH, Zhang DC (2023). Cryopreservation of black seabream (*Acanthopagrus schlegelii*) sperm. Theriogenology.

[24] Yuan XF, Wu JD, Liu BQ, Zhang YL, Wang XB (2005). Temperature control of the frozen Semen. Heilongjiang J. Anim. Reprod..

[25] Naer A (2020). Study on Cryopreservation of Mongolian Stallion Semen.

[26] Jha PK, Shahi Alam MG, Mansur AA, Naher N, Islam T, Uddin Bhuiyan M, Bari FY (2019). Cryopreservation of Bangladeshi ram semen using different diluents and manual freezing techniques. Cryobiology.

[27] Eriksson BM, Rodriguez-Martinez H (2000). Deep-freezing of boar semen in plastic film ‘cochettes’. Thai. J. Vet. Med..

[28] Prathalingam NS, Holt WV, Revell SG, Mirczuk S, Fleck RA, Watson PF (2006). Impact of antifreeze proteins and antifreeze glycoproteins on bovine sperm during freeze-thaw. Theriogenology.

[29] Küçük N, Aksoy M, Uçan U, Ahmad E, Naseer Z, Ceylan A, Serin I (2014). Comparison of two different cryopreservation protocols for freezing goat semen. Cryobiology.

[30] Motamedi-Mojdehi R, Roostaei-Ali Mehr M, Rajabi-Toustani R (2014). Effect of different levels of glycerol and cholesterol-loaded cyclodextrin on cryosurvival of ram spermatozoa. Reprod. Domest. Anim..

[31] Falchi L, Pau S, Pivato I, Bogliolo L, Zedda MT (2020). Resveratrol supplementation and cryopreservation of buck semen. Cryobiology.

[32] Nordstoga AB, Söderquist L, Adnøy T, Paulenz H (2011). Fertility results after vaginal deposition of frozen-thawed buck semen diluted with two different extenders using one- or two-step procedures. Reprod. Domest. Anim..

[33] Km M, Khalil MH, Al-Saef AM (2012). Effect of goat breeds, semen diluents and freezing methods on sperm freezability and reproductive performance. Assiut Vet. Med. J..

[34] Abush A, Hauser R, Paz G, Kleiman SE, Lehavi O, Yavetz H, Yogev L (2014). Thawed human sperm quality is influenced by the volume of the cryopreserved specimen. Fertil. Steril..

[35] Du C, Zheng X, Jiang J, Meng J, Wu Y, Gao X, Zhu J (2021). The effects of extenders, cryoprotectants and conditions in two-step cooling method on *Varicorhinus barbatulus* sperm. Cryobiology.

[36] Ruilan L (2018). Optimization of Cryopreservation of Inner Mongolian Cashmere Goats Semen and Study on the Action Mechanism of the Main Components of Semen Extender.

[37] Gilmore JA, Liu J, Woods EJ, Peter AT, Critser JK (2000). Cryoprotective agent and temperature effects on human sperm membrane permeabilities: Convergence of theoretical and empirical approaches for optimal cryopreservation methods. Hum. Reprod..

[38] Mazur P, Koshimoto C (2002). Is intracellular ice formation the cause of death of mouse sperm frozen at high cooling rates?. Biol. Reprod..

[39] Morris GJ, Faszer K, Green JE, Draper D, Grout BW, Fonseca F (2007). Rapidly cooled horse spermatozoa: Loss of viability is due to osmotic imbalance during thawing, not intracellular ice formation. Theriogenology.

[40] Yildiz C, Law N, Ottaviani P, Jarvi K, McKerlie C (2010). Comparison of sperm quality and DNA integrity in mouse sperm exposed to various cooling velocities and osmotic stress. Theriogenology.

[41] Agarwal A, Prabakaran S, Allamaneni S (2006). What an andrologist/urologist should know about free radicals and why. Urology.

[42] Lushchak VI (2014). Free radicals, reactive oxygen species, oxidative stress and its classification. Chem. Biol. Interact..

[43] Wang Y, Kang Y, Zhang L, Niu H, Sun X, Li Y (2022). Coenzyme Q10 improves the quality of sheep sperm stored at room temperature by mitigating oxidative stress. Anim. Sci. J..

[44] Ball B (2000). The effect of oxidative stress on equine sperm function, semen storage and stallion fertility. J. Equine Vet. Sci..

[45] Aitken JB, Naumovski N, Curry B, Grupen CG, Gibb Z, Aitken RJ (2015). Characterization of an L-amino acid oxidase in equine spermatozoa. Biol. Reprod..

[46] Zhao Y, Wang Y, Guo F, Lu B, Sun J, Wang J, Ren Z (2021). iTRAQ-based proteomic analysis of sperm reveals candidate proteins that affect the quality of spermatozoa from boars on plateaus. Proteome Sci..

